# Anti-Human Tissue Factor Antibody Ameliorated Intestinal Ischemia Reperfusion-Induced Acute Lung Injury in Human Tissue Factor Knock-In Mice

**DOI:** 10.1371/journal.pone.0001527

**Published:** 2008-01-30

**Authors:** Xiaolin He, Bing Han, Marco Mura, Li Li, Marcelo Cypel, Avery Soderman, Kristen Picha, Jing Yang, Mingyao Liu

**Affiliations:** 1 Latner Thoracic Surgery Research Laboratories, Department of Surgery, University Health Network Toronto General Research Institute, University of Toronto, Toronto, Ontario, Canada; 2 Therakos, Exton, Pennsylvania, United State of America; 3 Centocor Inc., Malvern, Pennsylvania, United States of America; Oregon Health & Science University, United States of America

## Abstract

**Background:**

Interaction between the coagulation and inflammation systems plays an important role in the development of acute respiratory distress syndrome (ARDS). Anti-coagulation is an attractive option for ARDS treatment, and this has promoted development of new antibodies. However, preclinical trials for these antibodies are often limited by the high cost and availability of non-human primates. In the present study, we developed a novel alternative method to test the role of a humanized anti-tissue factor mAb in acute lung injury with transgenic mice.

**Methodology/Principal Findings:**

Human tissue factor knock-in (hTF-KI) transgenic mice and a novel humanized anti-human tissue factor mAb (anti-hTF mAb, CNTO859) were developed. The hTF-KI mice showed a normal and functional expression of hTF. The anti-hTF mAb specifically blocked the pro-coagulation activity of brain extracts from the hTF-KI mice and human, but not from wild type mice. An extrapulmonary ARDS model was used by intestinal ischemia-reperfusion. Significant lung tissue damage in hTF-KI mice was observed after 2 h reperfusion. Administration of CNTO859 (5 mg/kg, i.v.) attenuated the severity of lung tissue injury, decreased the total cell counts and protein concentration in bronchoalveolar lavage fluid, and reduced Evans blue leakage. In addition, the treatment significantly reduced alveolar fibrin deposition, and decreased tissue factor and plasminogen activator inhibitor-1 activity in the serum. This treatment also down-regulated cytokine expression and reduced cell death in the lung.

**Conclusions:**

This novel anti-hTF antibody showed beneficial effects on intestinal ischemia-reperfusion induced acute lung injury, which merits further investigation for clinical usage. In addition, the use of knock-in transgenic mice to test the efficacy of antibodies against human-specific proteins is a novel strategy for preclinical studies.

## Introduction

One of the important features and major underlying mechanisms of acute lung injury (ALI) and its severe form acute respiratory distress syndrome (ARDS) is the intensive inflammatory response [1]. Evidence from animals and human studies indicates that alveolar and interstitial coagulation disorder and fibrin deposition are the hallmarks of early phase ALI/ARDS and other inflammatory situations in the lung, including pneumonia [2], sepsis [3], [4], and ventilator-induced lung injury [5], [6]. Coagulopathy not only adds another pathological feature to ALI/ARDS, but also leads to inflammatory signals through the activation of protease-activated receptors (PARs) [7], [8], [9], indicating an extensive cross-talk and reciprocal amplification between coagulation and inflammatory cascades [10].

Recently, increased attention has been paid in both experimental and clinical research of ALI/ARDS to the interplay between coagulation and inflammation [11], [12]. Anticoagulant therapies were developed and tested in animals and humans. An infusion of activated protein C showed a beneficial effect on the mortality in patients with severe sepsis [13], supporting the role of an anti-coagulant therapy to treat inflammation. Since tissue factor (TF) is a key initiator of the coagulation cascade and plays a critical role in inflammation as well, anti-TF therapy is another attractive anticoagulant strategy. Several reagents against the TF complex at sequential steps in its assembly, including a competitive inhibitor of Factor VIIa and an antibody to the Factor X binding site on TF, were tested in an *E. coli* sepsis model in baboons [4], [14], [15]. TF blockade has shown protective effects when administered at the onset of sepsis and given as a rescue therapy, decreasing systemic inflammation, preventing fibrinogen depletion, and attenuating injury to the lung, kidney, and other organs [4], [14], [15]. These promising results support further development of new molecules targeting coagulation pathways for clinical applications.

Currently, non-human primates are generally the best pre-clinical models to test efficacy of antibodies against human proteins. However, these models are often limited by their high cost and animal availability. In the present study, we tested a humanized anti-hTF monoclonal antibody (CNTO859) in a clinically relevant ALI model induced by intestinal ischemia-reperfusion (IIR). However instead of using non-human primates, we developed human TF knock-in (hTF-KI) transgenic mice, which express human TF instead of murine TF (mTF). CNTO859 treatment significantly reduced IIR-induced lung injury, attenuated alveolar fibrin deposition and inflammatory responses in the lung. This novel antibody and the transgenic approach against human genes to conduct pre-clinical studies in mice merit further investigations.

## Methods

### hTF-KI transgenic mice and anti-hTF monoclonal antibody, CNTO859

Generation of hTF-KI transgenic mice has been previously described [16]. Briefly, the first two exons of murine TF gene was replaced in-frame by human TF cDNA. As a result, expression of mTF was completely disrupted and hTF was expressed under the control of mTF promoter. Chimeric mice derived from targeted 129SvBrd embryonic stem cells were bred with C57BL/6 mice to produce hTF/mTF heterozygous mice and the breeding colony was established by further backcrossing with C57BL/6 mice. hTFKI homozygous mice (hTF/hTF) and wild type littermates used in this study were derived from breeding N2 heterozygous mice.

The anti-hTF monoclonal antibody, CNTO859, was genetically engineered by grafting the complementarity–determining regions of a murine anti-hTF antibody, TF8-5G9, onto a human immunoglobulin G4 frame. The antibody consists of 5–10% murine and 90–95% human proteins [17].

### Testing of hTF activity in transgenic mice

Pro-coagulant activity of hTF expressed in the transgenic mice was tested using a one-stage clotting assay with brain homogenate in comparison with that in wild type mice and in human brain tissue (ILSbio, Chestertown, MD). Approximately 25 mg brain tissues were homogenized in Hanks Balanced Salt Solution using Fast-prep protein isolator tubes (Bio 101 Systems, Qbiogene, Carlsbad, CA). Brain homogenates were frozen at −80°C immediately after isolation, and were diluted 1∶100 prior to use. The total protein was measured at OD_280 _to determine concentration. Citrated human plasma (100 µl) was incubated with increasing concentrations of brain homogenate (200 µl and 10 mM CaCl_2_) and the clotting time was recorded using Organon Teknika Coag-A-mate XM [18]. All samples were tested in duplicate. The data were fit to a hyperbolic curve to determine an EC_50_ (0.1 mg/ml for human and hTF-KI mouse brain extracts, 0.4 mg/ml for wild type littermate mouse extract).

We also used this assay to determine the inhibition efficacy and specificity of CNTO859 on human TF activity by incubating the brain extracts with increasing concentrations of either the CNTO859 or an anti-murine TF mAb. The changes of clotting time were recorded for comparison.

### Intestinal ischemia reperfusion-induced acute lung injury

Acute lung injury was induced by intestinal ischemia-reperfusion as previously described [19], [20] with modification. Briefly, male hTF-KI mice (6–8 weeks, 25–30 g) were anesthetized with 5% isoflurane. A tracheostomy cannula for mouse (H. Sachs Elektronik, March-Hugstetten, Germany) was inserted into the trachea, and animals were ventilated with a volume control ventilator (Inspira Advanced Safety Ventilator, Harvard Apparatus, St. Laurent, Canada) at a tidal volume of 6 ml/kg, inspiratory/expiratory ratio 1∶2 and a frequency of 140 breaths/min (FiO_2_ 100%). Anesthesia was maintained with 1.5% isoflurane and body temperature was maintained at 37°C throughout the experiment. A midline laparatomy was performed and the superior mesenteric artery was identified and occluded below the celiac trunk with an arterial micro-clamp (Mizuho Ikakogyo, Tokyo, Japan). Intestinal ischemia was confirmed by paleness of the jejunum and ileum. After 45 min of ischemia period, reperfusion was initiated by removal of the clamp and confirmed by the color recovery of the intestine. Pre-warmed (37°C) saline (0.5 ml) was instilled into the peritoneal cavity before closed with a suture. At 10 min after the onset of reperfusion, hTF-KI mice were randomized into 2 groups treated with either anti-hTF mAb (CNTO859, 5 mg/kg) or saline at same volume (2.5 ml/kg) through the jugular vein. Anesthesia and ventilation were terminated after treatment. All animals were left on spontaneous breathing during the 2 h of reperfusion, and then sacrificed by exsanguinations. The blood, lung and intestine tissues, and bronchoalveolar lavage (BAL) fluid were collected for further analysis. The experimental protocol was approved by the Toronto General Hospital Animal Care and Use Committee. All mice received care in compliance with the Principles of Laboratory Animal Care formulated by the National Society for Medical Research, and the Guide for the Care and Use of Experimental Animals formulated by the Canadian Council on Animal Care.

### Histology and immunostaining

A stretch of intestine from the middle of IR-challenged area and the right lungs were fixed in 10% formalin [20] and subjected for histological examination and immunostaining. Hematoxylin and eosin (H&E) staining was conducted with 5 µm tissue slides. The lung injury was assessed with modified scoring systems by a blinded pathologist according to the presence and extent of interstitial cellular infiltration, alveolar wall edema, hemorrhage, and atelectasis [21].

Immunostaining was performed for von Willebrand factor (vWF) in the lung tissue. Briefly, the lung sections at 5 µm thickness were blocked with 5% BSA (Sigma, Oakville, Canada) in PBS for 30 min at 37°C after deparaffinization and dehydration. The slides were then incubated with rabbit anti-vWF polyclonal antibody (1∶600 in 1% BSA, Dako, Mississauga, Canada) at 4°C overnight. ABC System (Vector Laboratories, Burlingame, CA) was used with a biotinylated goat anti-rabbit IgG (1∶200) as the secondary antibody and permanent red as the chromogen. The specificity of the antibody was determined by replacing the primary antibody with non-immunized IgG (Sigma).

### Cell counting and protein in BAL fluid

After ligation of the right bronchus, BAL was performed in the left lung by gently instilling and aspirating 0.25 ml saline through an intratracheal tube 2 times. An aliquot (20 µl) of BAL fluid from each animal was diluted with trypan blue (1∶1) for total cell counting with a hemocytometer. The rest of the fluid was centrifuged (4,000 g, 10 min), and a Bradford protein assay (Bio-Rad, Hercules, CA) was conducted for the protein concentration in the supernatant [20].

### Evans blue dye (EBD) assay and wet/dry (W/D) lung weight ratio

The lower lobes of the right lungs were collected and dehydrated at 60°C for 72 h in a vacuum oven. The wet and dry weights were measured to calculate the W/D ratio. The EBD assay was conducted as previously reported [20]. Evans blue dye (30 mg/kg, Sigma) was administered via jugular vein 30 min before the experiment terminated. After flushing with 10 ml of PBS, the extravasation of EBD in the lung tissues was extracted, and determined at 620 nm.

### Coagulation Assays

Activities of hTF and plasminogen activator inhibitor-1 (PAI-1) in the plasma were analyzed with a colorimetric assay according to the manufacturer's recommendations (American Diagnostica, Stamford, CT). Fibrin deposition was stained with Martius Scarlet Blue (MSB) using a standard protocol [22].

### Electron microscopy

After IIR challenge, fresh lung biopsies were taken for electron microscopy. The samples were fixed with 2% glutaraldehyde in 0.1 M sodium cacodylate buffer, post-fixed with 1% osmium tetroxide in the same buffer, dehydrated in graded ethanol series, and embedded in Spurr epoxy resin. The embedded tissues were thin-sectioned, mounted on copper grids, and stained with uranyl acetate and lead citrate, as previously described [19], [20], [23], [24]. Photographs were taken with an FEI CM100 Electron Microscope (FEI Company, Hillsboro, Oregon) equipped with a Kodak MegaPlus digital camera.

### Cytokine/chemokine measurement

Tumor necrosis factor α (TNFα), interleukin (IL)-6, IL-10, monocyte chemoattractant protein-1 (MCP-1), and Interferon-γ (IFN-γ) in BAL fluid and lung tissue homogenates were measured using a mouse inflammation kit of cytometric bead array according to the manufacture's instruction (BD Bioscience, Mississauga, Canada) [25]. In brief, an aliquot of 50 µl sample was incubated with 50 µl of mixed beads coated with capturing antibodies specific for the respective cytokines and 50 µl of PE-conjugated detection antibodies for 2 h at room temperature in dark. The beads were washed by adding 1 ml of wash buffer and centrifugation, and then re-suspended in 300 µl of wash buffer. The distinct fluorescence intensities of beads were determined with a flow cytometer, and the data acquired were converted to the concentrations of the cytokines using BD CBA software (Becton Dickinson).

### Terminal transferase dUTP nick end labeling (TUNEL) staining

The lung cell death was assessed by TUNEL staining with *In Situ* Cell Death Detection Kit (Roche, Penzberg, Germany) following the manufacture's instruction [20]. Briefly, after deparaffinization and dehydration the slides were permeabilized with 10 µg/ml proteinase K in 10 mM Tris/HCl (pH 7.4) for 15 min, and stained with Tetramethylrhodamine (TMR)-labeled TUNEL-positive nucleotides and counterstained with Hoechst (Pierce) for 10 min. Slides pre-treated with DNase (3,000 U/ml in 40 mM Tris-HCl, pH 7.5, 1 mg/ml BSA) were served as a positive control. Slides for negative control were incubated with the label solution without terminal transferase. The TUNEL-positive cells were quantified from 10 optical fields (400x) randomly chosen from each slide (n = 4 animals/group).

### Caspase 3 activity assay

Caspase 3 activity in the lung tissues was determined by measuring the fluorescence of cleaved caspase 3 substrate as described [19], [24], [26]. Lung homogenate containing 200 µg of total protein was mixed with 125 µM fluorogenic substrate (Ac-DEVD-AMC, Chemicon, Temecula, CA) in a 96-well plate. The plate was incubated at 37°C for 60 min and the fluorescence intensity (excitation of 360 nm and emission of 460 nm) was monitored with a CytoFluor multi-well plate reader (PerSeptive Biosystems Series 4000, Framingham, MA). The enzyme activity was calculated against a standard curve generated with recombinant caspase 3 (Chemicon). Tissue lysates from hypothermic preserved rat donor lungs [24] were used as positive controls for the assay.

### Statistical Analysis

Statistical software SPSS version 11.5 (SPSS, Chicago, IL) was used for data analyzing. Data are presented as mean±standard deviation (SD). All parametric data were analyzed with un-paired two-tailed *t*-test. Non-parametric data (lung injury scores) were analyzed with Kruskall-Wallis test. P value <0.05 is defined as significant.

## Results

### Human tissue factor maintains normal coagulant activity in transgenic mice

**Figure 1:**
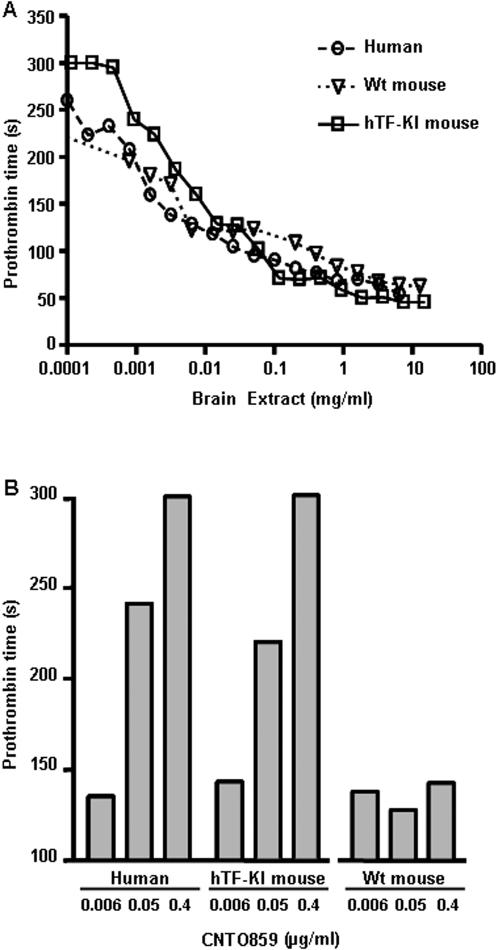
Human tissue factor is functionally expressed in hTF-KI transgenic mice, and effectively and specifically inhibited by the anti-hTF mAb, CNTO859. Pro-coagulant activity of TF in brain extracts from either hTF-KI or wild type (WT) mice, or from human brain tissue was measured with a one-stage clotting assay, and a similar prothrombin time was seen in all brain extracts, indicating a functional replacement of mTF by hTF expressed in the hTF-KI mice (A). Anti-hTF antibody, CNTO859, dose-dependently inhibited TF pro-coagulant activity in the brain extracts from hTF-KI mice and human, but not wild type mice (B). The experiments were repeated three times, and representative data from one experiment are shown.

To test anti-TF therapy against hTF in vivo, we used the hTF-KI mice generated by replacing a portion of mTF gene with the open reading frame of hTF. The hTF-KI mice have a similar life span without abnormal pathology in comparison with wild type animals. The tissue distribution and expression levels of hTF in transgenic mice were similar to that of mTF in wild type mice, and no mTF expression was detected in the transgenic mice [16]. The brain extract of hTF-KI mice expressed hTF at 64 ng/mg protein, a level comparable to that from the human brain extract at 80 ng/mg protein. Tested with a one-stage clotting assay, the brain extract from the hTF-KI mice showed a similar pro-coagulation activity as that from wild type mice or human brain tissue (Fig. 1A). These results suggest that hTF functionally substitutes mTF and maintains a normal coagulant activity in the hTF-KI mice.

When the humanized monoclonal antibody, CNTO859, was incubated with brain extracts from human or hTF-KI mice, it inhibited coagulation dose-dependently as measured by prothrombin time. The IC_50_ of CNTO859 for the hTF-KI brain extract was similar to that for the human brain extract (0.045 vs. 0.05 µg/ml), supporting that the hTF activity from both samples were comparable. In contrast, this antibody had no effect on brain extract from wild type mice (Fig. 1B). When an anti-mTF antibody was used in the coagulation assay, an inhibition effect was found only on brain extract from wild type mice, but not that from hTF-KI mice (data not shown). These results further confirm the specificity of CNTO859 on hTF and the substitution of mTF by the hTF is complete in the hTF-KI mice.

### Administration of CNTO859 ameliorated IIR-induced ALI

**Figure 2:**
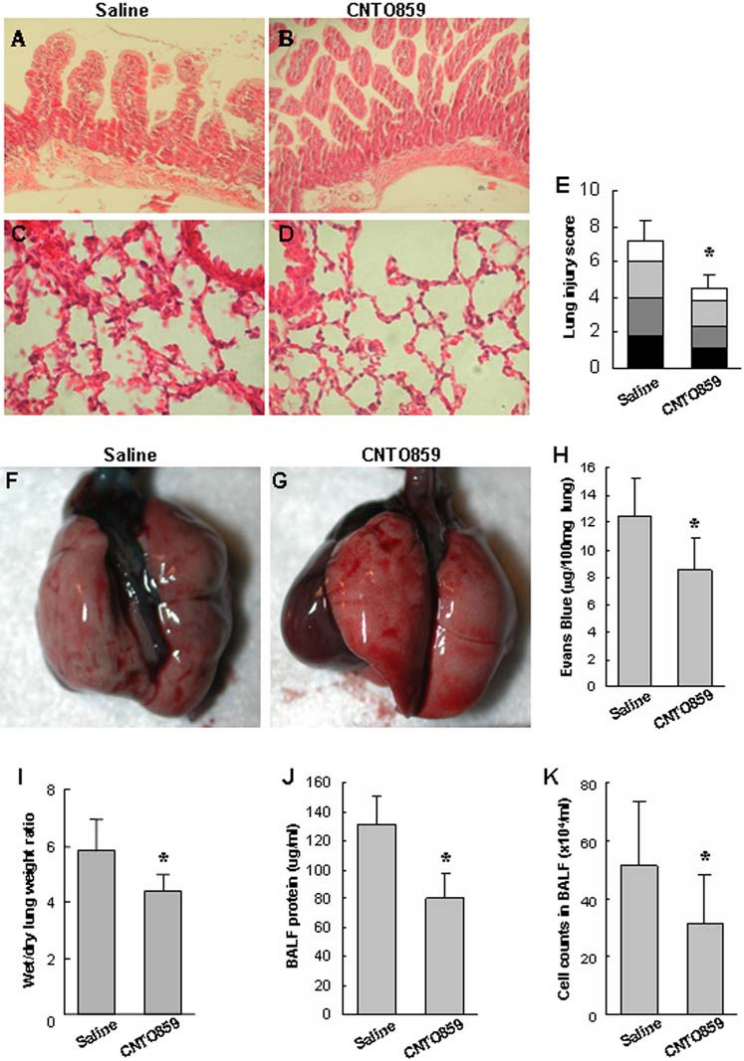
Administration of anti-hTF mAb ameliorated intestinal ischemia-reperfusion (IIR)-induced acute lung injury in hTF-KI mice. IIR challenged hTF-KI mice were treated with CNTO859 (5 mg/kg, i.v.) or saline. The representative histology (H&E, x400) from the intestine (A, B) and lung (C, D) was shown. The lung injury was scored by a pathologist in a blind fashion (E) (▪ inflammatory cells infiltration; ▪ alveolar wall edema; ▪ hemorrhage; atelectasis). □ Lung injury scores of 4 categories were analyzed with Kruskall-Wallis test, n = 4 animals/group, *: *p*<0.05. The pulmonary permeability was determined by Evans Blue dye assay (F, G, H). Administration of CNTO859 also reduced the wet/dry lung weight ratio (I), albumin concentration (J), and total cell counts (K) in the BAL fluid. Panels I-K: n = 4 animals/group,*: *p*<0.05, un-paired *t*-test.

A clinically relevant model of extrapulmonary ARDS has been used to induce acute lung injury by intestinal ischemia-reperfusion in C57BL/6 mice, and a high mortality rate was observed in these studies during 4 h of reperfusion period when animals were ventilated with oxygen [19], [20]. It is known that C57BL/6 mice are sensitive to hyperoxia [27]. In the present study, we used hTF-KI mice, which are of a hybrid strain of 129SvBrd (∼20%) and C57BL/6 (∼80%). We first conducted a pilot study with a modified protocol, in which animals were not subject to mechanical ventilation and pure oxygen during the reperfusion period. There was no mortality during the first 24 h of reperfusion (data not shown). The IIR challenge induced a significant intestinal (Fig. 2A) and lung injury (Fig. 2C) in hTF-KI mice, which are very similar to that in wild type mice (data not shown) and as observed in our previous study [19], [20]. These results suggest that hTF not only substituted mTF for coagulation, but also may play a similar role in acute inflammatory response related to acute lung injury.

We then used this modified IIR model to test the effects of anti-hTF antibody in acute lung injury. The IIR-induced ALI in the hTF-KI mice was characterized by increased pulmonary interstitial edema (alveolar wall thickening), inflammatory cell infiltration, hemorrhage, and atelectasis. Administration of anti-hTF antibody, CNTO859, markedly ameliorated the IIR-induced ALI (Fig. 2D) in hTF-KI mice with a significantly lower injury score (p<0.05, Fig. 2E).

One of the major features of ALI/ARDS is the increase in pulmonary permeability [28], [29]. A significant blockage of Evans Blue Dye leaking was seen in the lung treated with CNTO859 (Fig. 2F–H). This effect was confirmed with lung wet/dry weight ratio (Fig. 2I). The albumin content and total cell counts in the BAL fluid were significantly lower in CNTO859-treated group than in saline-treated group (Fig. 2J and 2K).

### Anti-TF mAb treatment attenuated IIR-induced coagulopathy

**Figure 3:**
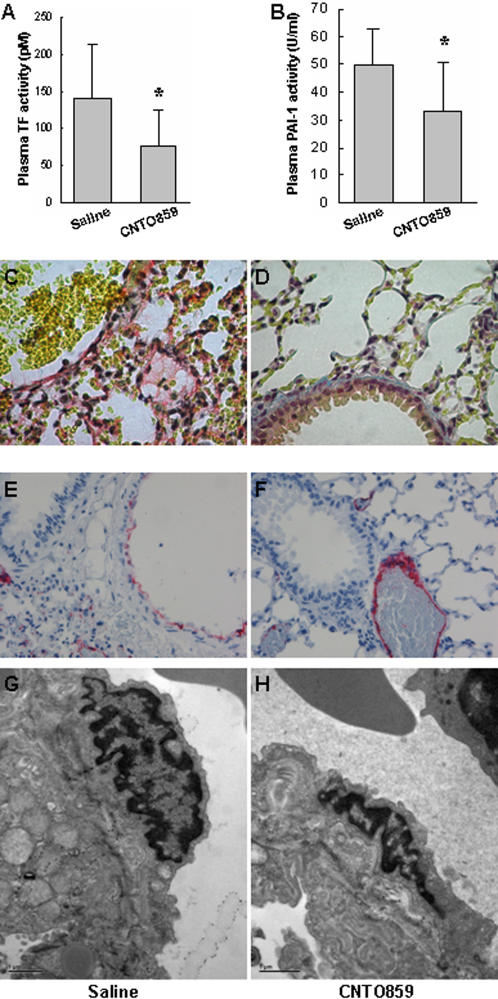
Anti-hTF mAb treatment attenuated IIR induced coagulapathy and protected pulmonary endothelium. Plasma TF and PAI-1 activities were determined as described in Methods. Administration of CNTO859 significantly inhibited both TF (A) and PAI-1 (B) activities in comparison with the saline treated control (*: *p*<0.05, n = 4 animals/group, un-paired t-test). The fibrin staining showed that CNTO859 ameliorated IIR-induced fibrin deposition (pink) in the alveoli (C, D). The lung tissues were stained for vWF, a specific marker for endothelial integrity. Weaker staining of vWF (pink) of endothelium in the pulmonary vessels was noted in the saline control group (E). In CNTO859 treated animals stronger vWF staining was observed in the endothelium of pulmonary vessels (F). The pulmonary endothelial injury was further examined with electron microscopy. IIR challenge led to significant endothelial cell swelling (G), which was protected by CNTO859 treatment (H).

It is known that activation of TF can trigger a pro-coagulation status, and lead to fibrin deposition in the lung. Administration of CNTO859 significantly reduced both TF and PAI-1 activities in the plasma (Fig. 3A and 3B), and dramatically attenuated the IIR-induced alveolar fibrin deposition (Fig. 3C and 3D).

Endothelium damage is an important mechanism responsible for the increase of pulmonary permeability [28], [29]. vWF is an endothelial specific marker, which is expressed mainly in larger vessels in normal lung tissue [30], [31]. In control animals, IIR challenge markedly reduced vWF immunostaining in larger pulmonary vessels (Fig. 3E). EM showed swelling of the cytoplasm, nucleus, and mitochondria of the endothelial cells in micro capillaries (Fig. 3G). In CNTO859 treated animals, vWF staining was clearly stronger in the endothelial layer of larger pulmonary vessels (Fig. 3F). The morphology of endothelial cells in pulmonary capillaries was better preserved (Fig. 3H).

### Anti-TF mAb treatment reduced inflammatory response and cell death in the lung

**Figure 4:**
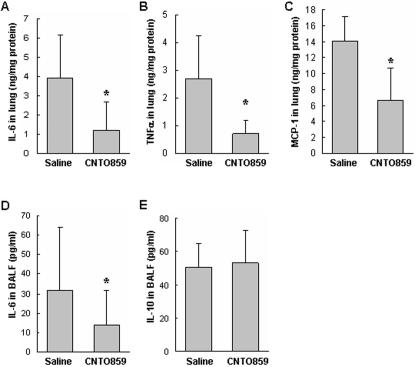
Anti-hTF mAb treatment reduced inflammatory response. Inflammatory cytokines were measured in the lung tissues and BAL fluid with a cytometric bead array. The expression levels of IL-6 (A), TNFα (B), and MCP-1 (C) in the lung tissues were significantly reduced by CNTO859 in comparison to saline group. In the BAL fluid CNTO859 also reduced the IL-6 levels (D), but the IL-10 level remained unchanged between the two groups (E). Un-paired t-test was used, *: *p*<0.05, n = 4 animals/group.

Acute inflammatory response is a hallmark of ALI. CNTO859 treatment significantly reduced the levels of IL-6 and TNFα, and MCP-1 in the lung tissue (Fig. 4A–C). The IL-6 levels in BALF (Fig. 4D) were significantly reduced by CNTO859. However, the levels of IL-10, an anti-inflammatory cytokine, did not change in BALF (Fig. 4E), suggesting that the anti-inflammatory effect of CNTO859 is not through up-regulation of IL-10.

**Figure 5:**
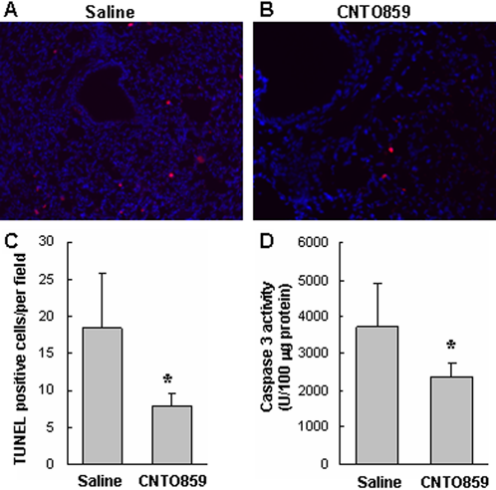
Anti-hTF mAb treatment reduced cell death. IIR-induced cell death in the lungs was determined by TUNEL staining. CNTO859 effect on the cell death was shown in representative slides (400x) (A, B), and quantified by counting the TUNEL positive cells from 10 randomly chosen fields (C). Caspase 3 activity in the lung tissue was also reduced by the anti-hTF antibody treatment (D). Un-paired t-test was used, 4 animals per group, *: *p*<0.05.

In a previous study, we found that IIR induces alveolar epithelial cell death [19]. In the present study, TUNEL positive cells were found in the lung of hTF-KI mice after IIR challenge (Fig. 5A), and they were significantly reduced by CNTO859 treatment (Fig. 5B and 5C). Caspase 3 is a key enzyme for apoptotic cell death. CNTO859 treatment reduced caspase 3 activity in lung tissue homogenates (Fig. 5D).

## Discussion

Using transgenic mice to test efficacy of antibodies raised against human proteins is an attractive and important concept. There are only a few reports of using this strategy, thus, the results from the present study provide more insights for further development of this novel and useful model methodology.

Tissue factor, also named as thromboplastin, CD-142, and coagulation factor III, is a single chain membrane glycoprotein that functions as a receptor for Factor VII and VIIa and thereby initiates the extrinsic pathway of the coagulation cascade in response to vascular injury [32], [33]. TF is generally localized in the adventitial fibroblasts of blood vessels, and initiates a rapid coagulation process when exposed to blood at the site of vessel injury, thereby limiting blood loss and maintaining hemostasis [34]. A compatibility study showed that hTF can bind to murine Factor VIIa with high affinity to induce coagulation [35], while mTF binds poorly to human Factor VIIa [35], [36]. Our hTF-KI transgenic mice showed expression of hTF at a physiological level that is fully compatible to normal lifespan, coagulation function [16] and similar inflammatory response upon IIR. The substitution or replacement of a murine protein with its human counterpart is crucial for evaluating human-specific therapeutic agents in murine models. The specificity and efficacy of the humanized monoclonal antibody is also critically important. The anti-hTF monoclonal antibody, CNTO859, specifically binds to hTF [17], and inhibits the hTF activation in brain extracts from human tissue and the hTF-KI transgenic mice, but not from wild type littermate mice. It should be mentioned that we used the antibody CNTO859 at a very high dose (20 mg/kg) in our preliminary experiments, and observed no significant haemorrhage in the treated animals, indicating the safety of the antibody (data not shown). These features are important for its potential clinical application in the future.

ARDS is a multi-factorial syndrome with similar pathological manifestations but distinct underlying mechanisms [1]. Anti-TF therapy is attractive not only because of TF's role in the coagulation cascade, but also because of its pivotal role in the interplay with inflammatory signaling. TF binding to Factor VIIa leads to activation of Factor X, forming a transient ternary complex, which activates coagulation, thus resulting in thrombin generation, and ultimately the clot formation. This complex has been demonstrated as being critical for presenting Factor VIIa and Factor Xa to PARs on the cell surface. Cleavage of PARs initiates the inflammatory rather than coagulant activities of TF, including upregulation of cytokine gene expression [7], [9], [14]. The requirement of TF as a co-receptor for PAR1 and PAR2 activation is likely to be a key determinant [37]. Recently, evidence showed the cytoplasmic domain of TF is also involved in chemotaxis regulation [38].

In the present study, we used IIR to induce ALI in hTF-KI mice. This is a clinically relevant model in which the severe intestinal damage is the initial insult. The lung is the most vulnerable remote organ after IIR, although cell death and inflammation have been noted in other vital organs [20]. This model has added value to other studies related to anti-TF therapies. Our data show a significant attenuation of the lung injury induced by IIR. The pulmonary permeability barrier consists of capillary endothelial and alveolar epithelial cells. Damage could occur on both sides of the alveolar walls during ALI [39]. In the present study, administration of anti-hTF antibody not only protected pulmonary endothelium from severe injury, but also significantly reduced cell death in the lung tissue, which mainly happens in alveolar epithelial cells upon IIR challenge [20]. Thus, anti-TF therapy may have protective effects on both endothelial and epithelial layers of alveolar walls. Ideally, a humanized IgG, instead of normal saline should be used for comparison with CNT0859, to exclude non-specific IgG effects. Our studies were limited by the availability of this agent; the results should be interpreted with caution.

The pulmonary level of TF expression has been found especially high relative to other organs. The TF levels in pulmonary edema fluid were found to be more than 100-fold higher than that in the plasma in patients with ALI/ARDS, indicating a local hypercoagulation status and tissue damages in the lung. The TF expression and activity in lung alveolar epithelial cells was increased by pro-inflammatory cytokines (e.g. TNFα and IL-1ß) [40]. We speculate that local administration of an anti-TF antibody may have direct benefits to ameliorate ALI, especially injury induced by intrapulmonary insults, such as acid aspiration and lung transplantation.

The hTF-KI animals and the specific antibody may offer us a useful model system to better characterize and understand the effects of anti-TF therapy in ALI models induced by other insults, such as sepsis, ventilator-induced lung injury, bacterial infection, etc. Results from these studies may provide additional information about the role of TF, as well as the potential therapeutic efficacy of CNTO859. Using small animals, we will be able to collect critical data prior to studies in non-human primates and clinical trials. This strategy should be considered for the development of species-specific therapeutic reagents for ALI/ARDS and other human diseases.

In summary, this study demonstrated the potential therapeutic effects of anti-TF strategy on ALI/ARDS with a new anti-TF monoclonal antibody. The new experimental strategy of using humanized antibody against human TF in transgenic mice offers a useful tool for further assessing the anti-TF therapy in preclinical trials for ARDS as well as other diseases related to the function of tissue factor.
